# Relationships among nursing students’ self-concept clarity, meaning in life, emotion regulation ability and depression: Testing a moderated mediation model

**DOI:** 10.3389/fpsyg.2022.1003587

**Published:** 2022-11-29

**Authors:** Youjuan Hong, Xuan Zhang, Weiwei Wu, Jingjing Chen, Yan Lin, Junyu Zhao, Huimin Xiao

**Affiliations:** School of Nursing, Fujian Medical University, Fuzhou, Fujian Province, China

**Keywords:** self-concept clarity, meaning in life, emotion regulation ability, depression, nursing students

## Abstract

**Objective:**

Self-concept clarity as an inducing factor of depressive symptoms has been confirmed in previous studies. However, little is known about how and when it induces depressive symptoms in nursing students. The study is to examine the potential mediating role of meaning in life and the moderation of emotion regulation ability in the association between self-concept clarity and depressive symptoms among college nursing students.

**Materials and Methods:**

A sample of 488 college nursing students participated in this study Southeast China. The Chinese adaptations of Self-concept clarity Scale, Sense of life Scale, Depression scale, Emotion regulation scale were used. Mediation and moderation analyses were carried out in the SPSS macro PROCESS.

**Results:**

Self-concept clarity was significantly and negatively associated with depression in nursing students, meaning in life had a partial mediating effect on the relationship between self-concept clarity and depression. Furthermore, emotion regulation ability moderated the direct effect of self-concept clarity on depression.

**Conclusion:**

The findings enrich the knowledge of the mediating and moderating mechanisms to explain the association between self-concept clarity and depression in nursing students. There have been proposed interventions concerning increasing self-concept clarity, meaning in life and emotion regulation ability training which could help help reduce the depression among nursing students.

## Introduction

Depression is the most serious mental health problem affecting undergraduate students, among whom rates of major depressive disorder range from 5.6 to 20% (Othieno et al., 2014). Previous research found that mental health problems, especially depression, are common in nursing students (Chernomas and Shapiro, 2013; Zeng et al., 2019; Mcdermott et al., 2020). For instance, nursing students reported to experience higher levels of stress, anxiety and depression than do the general student body (Goff, 2011; Van der Riet et al., 2015; Bartlett et al., 2016; McConville et al., 2017). Depression can not only lead to problems such as poor mood, decreased attention, slow thinking, and physical and sleep disorders, it can also cause problems with nursing students’ interpersonal communication, academic engagement, clinical practice, and social adaptation, and even lead to self-injury and suicidal behavior (Aloufi et al., 2021). Therefore, exploring the influential factors and mechanism of depression have important practical significance for the prevention of and interventions for psychological problems in students of nursing.

However, current research has focused more on the negative effects of stress perception and occupational identity on depression in nursing students, and less on improving the effects of general positive psychological characteristics on their mental health. In recent years, the psychological characteristics related to self-concept clarity have attracted academic attention. Self-concept clarity plays an important role of psychological adjustment. Higher self-concept clarity was found to correlated with different positive outcomes, such as lower levels of anxiety and depression (Butzer and Kuiper, 2006; Wong et al., 2019). Although researchers have increased their focus on self-concept clarity, little is known about the inner mechanism of the relationship between self-concept clarity and depression in nursing students. Thus, it is necessary to address mental health in nursing students from the aspect of self-concept clarity because such individuals are future professional care givers. To address these gaps in the literature, the present study explored whether meaning in life plays the mediation role of the links between self-concept clarity and depression and whether emotion regulation ability moderated the mediation model.

### Self-concept clarity and depression

The cognitive theory of depression suggests that the way people view themselves and process personal information contribute to the disease, especially individuals’ negative views of themselves (Pössel and Smith, 2020). As an important intrapersonal resource, self-concept clarity refers to the extent to which individuals have a clear and coherent sense of their own personal identity, a component of the self (Campbell et al., 1996). Previous studies have found self-concept clarity to be negatively associated with depression (Parise et al., 2019). Individuals holding a stable and consistent view of themselves tend to have higher well-being. Conversely, individuals lacking clarity in their sense of self tend to be more anxious and depressed (Bigler et al., 2001) and less satisfied. Improved self-concept clarity is also related to a variety of positive outcomes, such as higher self-esteem (Wu et al., 2010), while low self-esteem is an important risk factor for depression (Sowislo and Orth, 2013). In addition, individuals with low levels of self-concept clarity are more likely to view the world as chaotic, unpredictable, and stressful, and they may be more sensitive and vulnerable and susceptible to the effects of depression. Thus, the following hypothesis is proposed:

*Hypothesis 1*: Self-concept clarity negatively predicts depression in nursing students.

### Meaning in life as a mediator

Meaning in life is defined as “the sense made of, and significance felt regarding, the nature of one’s being and existence” (Steger et al., 2006). An individual with high presence of meaning (versus one who is searching for meaning) has a stronger association with psychological well-being (Jin et al., 2016; Van Tongeren et al., 2017). According to theory of meaning in life, those with self-concept clarity promote meaning in life by helping people organize their fragmented daily experiences and integrate new practices (Shin et al., 2016). Previous research found that self-concept clarity positively correlated with meaning in life (Nie and Gan, 2017). Individuals with high self-concept clarity tend to realize their own value, and make positive attributions in the face of stressors, thus experiencing more meaning in life (Nie and Gan, 2017). Nursing students often struggle with academic stress because of the demanding curriculum, excessive amount of information, and rigors of clinical training (Jeong and Shin, 2006). Self-concept clarity may help nursing students feel less vulnerable to being negatively affected by challenging situations.

Previous research has found meaning in life negatively correlated with anxiety and depression (Korkmaz and Güloğlu, 2021; Tsibidaki, 2021). Meaning in life manifests in the positive and powerful functions of psychological repair and construction. Individuals with high meaning in life tend to experience a greater sense of happiness (Krok, 2018), have superior performance and attain more achievement in their professional and social fields (Stillman et al., 2011). Conversely, the emptiness and boredom caused by a lack of meaning in life can lead to depression, irritability, post-traumatic stress disorder, and drug dependence. It has further been shown that meaning in life mediated the relationship between peer bullying and the individual internalization of problems (e.g., depression, anxiety; Henry et al., 2014). Thus, the following hypothesis is proposed:

*Hypothesis 2*: Meaning in life plays a mediating role in the effect of self-concept clarity on depression.

### Emotion regulation ability as a moderator

The effect of self-concept clarity on nursing students’ likelihood of depression may also be moderated by other factors. Previous studies have found that the ability to regulate emotions plays a moderating role in depression (Strauman and Eddington, 2017; Ottenstein, 2020). Emotion regulation is defined as the process influencing how and when individuals experience and express certain emotions, indicating one’s ability to manage emotional experiences and expressions (Gross and Jazaieri, 2014). Individuals with less ability to regulate their emotions tend to have difficulties avoiding depression when faced with stress, whereas individuals with greater emotion regulation ability tend to reduce the negative effects of emotional events (Joormann and Stanton, 2016).

The protective factor of depression model argues that the presence of one protective factor (such as emotion regulation) can enhance the role of other protective factors (e.g., self-concept clarity; Wang, 2012). Self-concept clarity play a protective role against depression (Lee-Flynn et al., 2011). Compared with individuals with higher levels of self-concept clarity, those with lower levels tend to be more likely to experience depression (Lee-Flynn et al., 2011). On the condition of high emotional regulation ability, the protective effect of self-concept clarity on depression may be relatively strong. Thus, the following hypothesis is proposed:

*Hypothesis 3*: Emotion regulation moderates the link between self-concept clarity and depression.

### The present study

**Figure 1:**
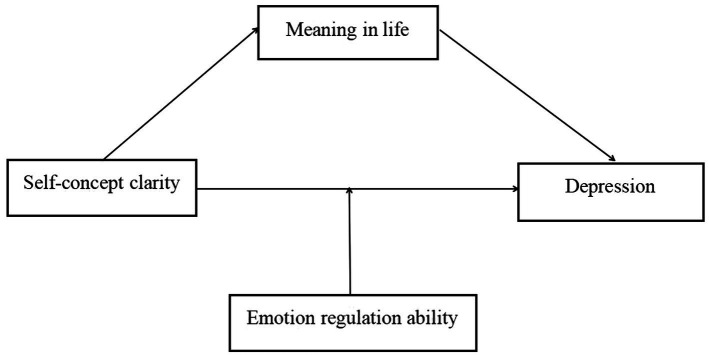
Proposed theoretical model.

In summary, this present study constructed a moderated mediation model to explore the inner mechanisms of self-concept clarity predicting the nursing students’ depression, to provide ideas for preventing and relieving depression in nursing students (Figure 1). The results will contribute to the current literature by extending our understanding of the mechanism that connects self-concept clarity and depression.

## Materials and methods

### Participants

Using the convenient cluster sampling method, nursing students from two medical universities in the Southeast China were selected to receive a questionnaire survey. A total of 510 questionnaires were distributed. Out of these, 22 questionnaires, which were not properly filled out, were dropped; finally, we were left with 488 valid samples. The effective rate of questionnaire recovery was 95.68%. Respondents’ average age was 19.88 years (*SD* = 1.32) and the group included 421 females (86.27%) and 67 males (17.33%).

### Measures

#### Self-concept clarity scale

Self-concept clarity was measured *via* The Self-Concept Clarity Scale developed by Campbell et al. (1996) and revised by Niu et al. (2016). It consists of 12 items rated on a four-point Likert scale (1 = strongly disagree, 4 = strongly agree). SampNiule items include: “I seldom experience conflict between the different aspects of my personality” and “In general, I have a clear sense of who I am and what I am.” Higher scores indicate a greater degree of self-concept clarity. The scale has been found in previous studies to have a high internal consistency and criterion validity (Vartanian and Dey, 2013). In the current research, the Cronbach’s alpha of the scale was 0.85.

#### Sense of life questionnaire

Meaning in life was measured *via* the Sense of Life Questionnaire compiled by Steger et al. (2006) and revised by Liu and Gan (2010). It has two dimensions, the search for and existence of meaning in life. The subscale of existence of meaning in life was used in the current study. The scale consists of five items, each rated on a seven-point scale (1 = definitely not true, 7 = definitely true). Higher scores indicate a greater degree of meaning in life. The scale has been found in previous studies to have a high internal consistency and criterion validity (Yen, 2014). In the current study, the Cronbach’s alpha of the scale was 0.90.

#### Depression scale

Depression was measured using the Chinese version of Zung’s Self-Rating Depression Scale (Zung, 1965). It consists of 20 items, each of which requires responses to be recorded on a rating scale (1 = rarely or never, 4 = most of the time or always). Sample items include “I do not sleep well at night.” Higher scores indicate a greater degree of depression. The scale has been found in previous studies to have a high internal consistency and criterion validity (Martínez et al., 2020). In the current study, the Cronbach’s alpha of the scale was 0.82.

#### Emotion regulation scale

Emotion regulation ability was measured *via* Emotional Regulation Scale compiled by Law et al. (2004). The scale has four items. Sample items include “I’m very good at controlling my emotions.” All items were rated on a seven-point Likert scale (1 = strongly disagree, 7 = strongly agree). Higher scores indicate a greater degree of emotion regulation ability. In the current study, the Cronbach’s alpha of the scale was 0.73.

#### Procedure

This investigation was approved by the Ethics Committee at the first authors’ institution. We obtained consent from all participants. Nursing students were invited to complete the questionnaires anonymously and free to withdraw from the study at any time. Due to the time, cost, and accessibility factors, the convenience sampling method is used as this method provides the highest response level while saving resources and timely feedback (Etikan et al., 2016).

### Data analysis

Data analyses were conducted *via* SPSS 21. Data collected by the self-report method may lead to common method deviation. Harman’s single-factor test was used to assess the possibility of common method bias; no common method variance was detected (20.22% interpretation rate of the first factor <40%; Lee et al., 2011). We used Models 4 and 5 of the PROCESS macro for SPSS to test the mediation and moderated mediation models, with 5,000 random sample bootstrapping confidence intervals (Hayes, 2017). All variables were standardized prior to being analyzed. According to previous research (Díaz-Narváez et al., 2020), the original data were combined into a single data base and analyzed using normality tests (Kolmogorov–Smirnov, for data greater than or equal to 50) and homoscedasticity (Levene).

## Results

### Preliminary analyses

The means, standard deviations, and Pearson’s correlations are presented in Table 1. Self-concept clarity was positively correlated with meaning in life and emotion regulation ability, but negatively correlated with depression. Meaning in life was positively correlated with emotion regulation ability and negatively correlated with depression. Emotion regulation ability was negatively correlated with depression. The normality tests and homoscedasticity were not significant (*p* > 0.05) and it was inferred that the data had a normal distribution and equality of variance between the groups compared. As a result, the relevant parametric statistical tests could be used.

**Table 1:** Descriptive statistics.

	*M*	*SD*	1	2	3	4	5	6
1. Gender	-	-	1					
Grade	-	-	0.09	1				
3. Self-concept clarity	2.41	0.39	0.01	0.01	1			
4. Meaning in life	4.58	1.07	0.07	−0.02	0.15***	1		
5. Emotion regulation ability	4.63	1.22	0.01	−0.07	0.14**	0.47***	1	
6. Depression	2.06	0.44	−0.09	−0.01	−0.29***	−0.45***	−0.39***	1

*M*, mean; *SD*, standard deviation. *p < 0.05, **p < 0.01, ***p < 0.001.

### Analysis of meaning in life as a mediator

**Figure 2:**
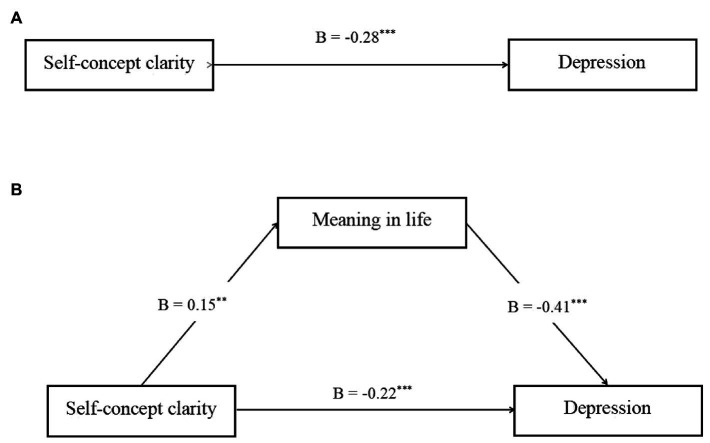
Mediator model depicting direct and indirect effects of self-concept clarity on depression tested in the current study. Graphic **(A)** depicts the total effect of self-concept clarity on depression. Graphic **(B)** depicts the direct effect of self-concept clarity on depression after including mediators. *p < 0.05, **p < 0.01, ***p < 0.001.

Model 4 of the PROCES macro (Hayes, 2017) was used to test the mediating effect of meaning in life (see Figure 2). The results showed that self-concept clarity significantly negatively predicted depression (*B* = −0.22, *р* < 0.001) and significantly positively predicted meaning in life (*B* = 0.15, *р* < 0.001); meaning in life significantly negatively predicted depression (*B* = −0.41, *p* < 0.001). However, the residual direct effect remained significant, showing that meaning in life mediated the relationship between self-concept clarity and depression (indirect effect = −0.07, 95% CI = −0.12 to −0.02). The total mediating effect (−0.07) accounted for 24.13% of the total effect (−0.32). This finding supported Hypotheses 1 and 2.

### Analysis of emotion regulation ability as a moderator

Model 5 of the PROCES macro (Hayes, 2017) was used to test the hypothesis regarding the moderating effect of emotion regulation ability (see Table 2). The interaction terms of “self-concept clarity × emotion regulation ability” being significant served as evidence of emotion regulation ability’s moderating effect (Frazier et al., 2004). The results showed that self-concept clarity predicted meaning in life (*b* = 0.15, 95% CI = [0.06, 0.23], *t* = 3.35, *p* < 0.01). The estimated coefficient indicated that meaning in life was also a predictor of depression (*b* = −0.31, 95% CI = [−0.40, −0.23], *t* = −7.30*, p* < 0.01), and the interaction term for self-concept clarity and emotion regulation ability was significant (*b* = −0.08, 95% CI = [−0.15, −0.01], *t* = −2.36*, p* < 0.01). Thus, the results supported Hypothesis 3.

**Table 2:** Coefficients for the tested moderated mediation model (*N* = 488).

	*R* ^2^	*F*	Coeff.	SE	95% CI
Meaning in life	0.03	6.63			
Constant			−0.31	0.25	−0.79 to 0.16
Self-concept clarity			0.15***	0.04	0.06 to 0.23
Depression	0.30	40.82			
Constant			0.08	0.21	−0.33 to 0.49
Self-concept clarity			−0.21***	0.04	−0.28 to −0.12
Meaning in life			−0.31***	0.04	−0.40 to −0.23
Emotion regulation ability			−0.20***	0.04	−0.29 to −0.12
Self-concept clarity × Emotion regulation			−0.08**	0.03	−0.15 to −0.01

SE, standard error; 95% CI, confidence interval with lower and upper limits; *p < 0.05; **p < 0.01; ***p < 0.001.

**Figure 3:**
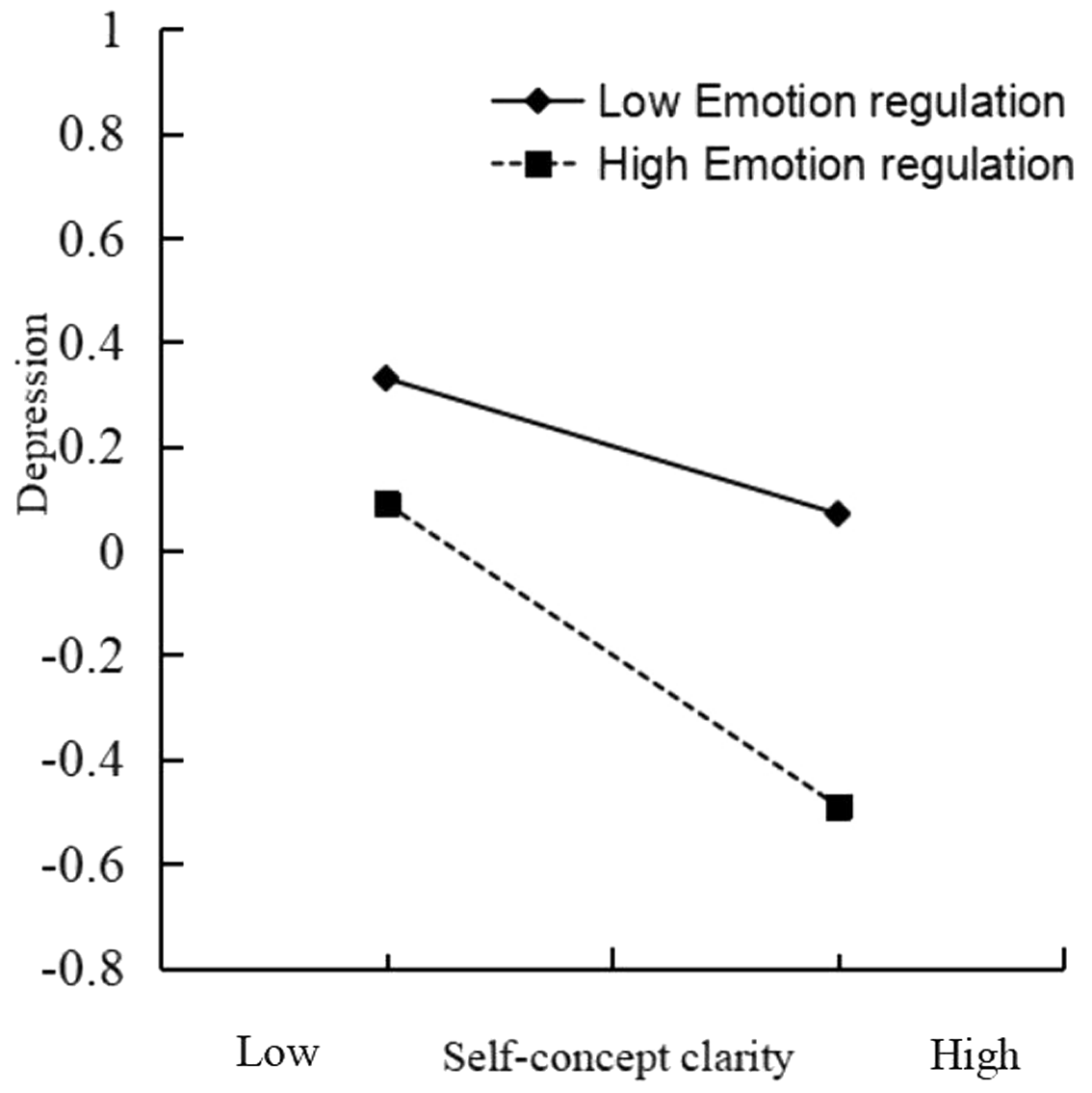
Interaction between self-concept clarity and emotion regulation ability.

A simple slope analysis (see Figure 3) was conducted to determine the separate relationships between self-concept clarity and depression. The results showed that for participants with lower levels of emotion regulation ability (*m* - 1sd), self-concept clarity had a significant negative effect on depression: simple slope = −0.12, *t* = −2.43, *p* < 0.05. For participants with higher regulation ability (*m* + 1sd), self-concept clarity had a greater negative effect on depression: simple slope = −0.28, *t* = −5.62, *p* < 0.001. This indicated that with improvements in nursing students’ emotion regulation ability, the negative predictive effect of self-concept clarity on depression showed a gradual upward trend.

## Discussion

Self-concept clarity is believed to be a critical characteristic of mental health. However, there is a dearth of research addressing the possible mediators and moderators that might explain the inner mechanism of nursing students. To address these issues, based on the cognitive theory of depression, meaning in life theories, and protective factor of depression model, the present study constructed a mediated moderation model that not only explained how self-concept clarity affects nursing students’ likelihood of depression (mediation of meaning in life), but also answered the question of when self-concept clarity might have a more significant effect on depression (moderation of emotion regulation ability). To our knowledge, this is the first study to investigate the role of meaning in life and emotion regulation in the association between self-concept clarity and depression in nursing students.

### The relationship between self-concept clarity and depression

The present study extended the research on self-concept clarity in nursing students. Although the link between self-concept clarity and depression is established in the literature (Lee-Flynn et al., 2011), the mechanism underlying this association is still not clear. As expected, also in our sample, nursing students with higher levels of self-concept clarity had lower levels of depression, which is consistent with previous research (Seo et al., 2020). This result supports the cognitive theory of depression. Nursing students with high levels of self-concept clarity have a clear and consistent understanding of themselves, and their self-esteem levels are higher, which can effectively buffer the impact of negative emotional experiences. Nursing students with low self-concept clarity can become confused about what kind of person they are and may be more vulnerable to various stressors, leading to the occurrence of depression. Therefore, having a coherent self-understanding should be crucial for depression and increasing self-concept clarity in nursing students may protect them from pressures and help them persist in adverse situation. Nursing educators could design and operate interventions that commonly emphasize self-knowledge and include several strategies or activities related to exploration and articulation of various aspects of self.

### Mediation of meaning in life

This study found that self-concept clarity significantly predicted meaning in life, a result consistent with those of previous studies (Shin et al., 2016). The finding that a sense of meaning in life mediates the relationship between self-concept clarity and depression supports meaning in life theories. The result points that self-concept clarity is an important factor for nursing students to gain a sense of meaning in their lives. Specifically, nursing students with high self-concept clarity have a more complete self-understanding and thus can deeply understand meaning in life (Błażek and Besta, 2012), find goals that contribute to self-actualization, and ultimately have a higher sense of meaning in life (Schlegel et al., 2009). The higher nursing students’ self-concept clarity was the more they were able to manage negative emotional states in response to stressful situations. Self-concept clarity and meaning in life are protective factors against depression in nursing students. Meaning in life allows nursing students to cope with stress and bounce back in the face of adversity (Cheng et al., 2021). Therefore, activities for increasing meaning in life seem to be one of the promising ways to decreasing depression among nursing students. Nurse educators may consider ways of emphasizing the importance of positive emotion through meaning-oriented programs and mental health classes.

### Moderation of emotion regulation ability

This study found that emotion regulation ability played a moderating role in the relationship between self-concept clarity and depression. Among nursing students with a high level of emotion regulation ability, higher self-concept clarity was significantly associated with lower depressive symptoms (Parise et al., 2019). However, this relationship became weaker in nursing student with a low level of emotion regulation ability. In other words, a high level of emotion regulation ability can mitigate the effect of low self-concept clarity on nursing students’ depression (Wang et al., 2021). This result is consistent with the “protective-protective factor” model. Nursing students with high levels of emotional regulation can overcome negative emotions such as anxiety, fear, and worry that are caused by self-confusion (Merino-Tejedor et al., 2016). Therefore, emotion regulation promotes the effect of self-concept clarity on depression among nursing students, Lower levels of self-concept clarity resulted in more depression, and therefore, higher levels of emotion regulation reduced the effect of self-concept clarity on depression in nursing students. Nursing educators should emphasize that emotion management training can be used as an effective tool in solving emotional distress.

### Limitations

Although this study examined in depth the mechanism by which self-concept clarity influences depression among nursing students, helping to deepen the understanding of the relationship between the two, there were several shortcomings that need to be resolved in future research. Firstly, the participants were recruited by convenience sampling, potentially leading to significant selective bias. A randomized sampling study should be conducted in the future. Secondly, in the current study, female undergraduates constituted the majority of the sample, decreasing the generalizability of the findings to a more representative population. The findings of the current study were limited to the nursing students from two medical universities, it may be difficult to generalize the results beyond the samples. Therefore, more male students from more universities should be investigated in the future.” Thirdly, the cross-sectional design does not allow confirmation of causal inferences about the association between the independent variables and depression. Future research should be combined with a longitudinal study to explore this in further depth.

## Conclusion

The current findings indicated that the relationship between self-concept clarity and depression can be mediated by meaning in life. Furthermore, the results revealed that emotion regulation ability moderated the negative effect of self-concept clarity on nursing student’s depression. The result indicated the critical role of self-identity clarity, meaning in life and emotion regulation ability for positively adjusting to the developmental challenges among nursing students. The study highlights three characteristics for nursing student’s mental health that nursing educators may need to consider: building/enhancing self-concept clarity; increasing meaning in life, and cultivating emotion regulation ability.

## Data availability statement

The raw data supporting the conclusions of this article will be made available by the authors, without undue reservation.

## Ethics statement

The studies involving human participants were reviewed and approved by the Academic Ethics Committee of Fujian Medical University. The patients/participants provided their written informed consent to participate in this study.

## Author contributions

YH contributed to the conceptualization, original draft-writing, and data curation. XZ, WW, YL, JC, and JZ contributed to the formal analysis. HX contributed to the writing - review and editing. All authors contributed to the article and approved the submitted version.

## Conflict of interest

The authors declare that the research was conducted in the absence of any commercial or financial relationships that could be construed as a potential conflict of interest.

## Publisher’s note

All claims expressed in this article are solely those of the authors and do not necessarily represent those of their affiliated organizations, or those of the publisher, the editors and the reviewers. Any product that may be evaluated in this article, or claim that may be made by its manufacturer, is not guaranteed or endorsed by the publisher.
